# ITGA5 Is a Novel Oncogenic Biomarker and Correlates With Tumor Immune Microenvironment in Gliomas

**DOI:** 10.3389/fonc.2022.844144

**Published:** 2022-03-18

**Authors:** Shuyu Li, Nan Zhang, Shiyang Liu, Hao Zhang, Jiajing Liu, Yiwei Qi, Qi Zhang, Xingrui Li

**Affiliations:** ^1^ Department of Thyroid and Breast Surgery, Tongji Hospital, Tongji Medical College, Huazhong University of Science and Technology, Wuhan, China; ^2^ One-Third Lab, College of Bioinformatics Science and Technology, Harbin Medical University, Harbin, China; ^3^ Department of Neurosurgery, Xiangya Hospital, Central South University, Changsha, China; ^4^ Department of Neurology, Affiliated People’s Hospital, Fujian University of Traditional Chinese Medicine, Fuzhou, China; ^5^ Department of Neurosurgery, Tongji Hospital, Tongji Medical College of Huazhong University of Science and Technology, Wuhan, China; ^6^ Department of Plastic and Cosmetic Surgery, Tongji Hospital, Tongji Medical College of Huazhong University of Science and Technology, Wuhan, China

**Keywords:** glioma, ITGA5, tumor microenvironment, immune cells (ICs), immune checkpoint

## Abstract

Gliomas are the most aggressive primary intracranial malignancies with poor overall survival. ITGA5 is one member of the integrin adhesion molecule family and is implicated in cancer metastasis and oncogenesis. However, few studies have explored the association between tumor immune microenvironment and ITGA5 expression level in gliomas. Firstly, we analyzed 3,047 glioma patient samples collected from the TCGA, the CGGA, and the GEO databases, proving that high ITGA5 expression positively related to aggressive clinicopathological features and poor survival in glioma patients. Then, based on the ITGA5 level, immunological characteristics and genomic alteration were explored through multiple algorithms. We observed that ITGA5 was involved in pivotal oncological pathways, immune-related processes, and distinct typical genomic alterations in gliomas. Notably, ITGA5 was found to engage in remolding glioma immune infiltration and immune microenvironment, manifested by higher immune cell infiltration when ITGA5 is highly expressed. We also demonstrated a strong correlation between ITGA5 and immune checkpoint molecules that may be beneficial from immune checkpoint blockade strategies. In addition, ITGA5 was found to be a robust and sensitive indicator for plenty of chemotherapy drugs through drug sensitivity prediction. Altogether, our comprehensive analyses deciphered the prognostic, immunological, and therapeutic value of ITGA5 in glioma, thus improving individual and precise therapy for combating gliomas.

## Introduction

A glioma is the most frequent central nervous system malignancy with an annual incidence of about 7 cases per 100,000 people (1). Glioma patients usually present neurological symptoms such as headache, pain, weakness, mood change, seizures, and loss of physical function and cognitive function. Despite the vast advancements of surgery, standard chemoradiotherapy, and adjuvant therapies, glioma patients are still confronted with fatal prognoses (2). It is intensively acknowledged that glioma is a highly heterogeneous tumor entity accompanied by multiple molecule characteristics, which could significantly impact clinical outcomes. For example, the status of O-6-methylguanine-DNA methyltransferase (MGMT) promoter methylation is a prognostic biomarker in glioma patients treated by temozolomide, but showing a minimal benefit for elderly patients (3). Nowadays, there is an urgent need to identify novel and reliable prognostic biomarkers to improve the clinical outcomes of glioma.

Glioma is an immunosuppressive tumor that encompasses a complicated tumor microenvironment (TME) comprising various cellular components, namely, glioma cells, stromal cells, and immune cells. The most abundant immune cells are tumor-associated macrophages (TAMs), which play an essential role in helping glioma proliferation, invasion, chemoradiotherapy resistance (4). Generally speaking, a glioma is a specific tumor type in that the blood–brain barrier (BBB) establishes a naturally occurring “immunologically privileged” site (5). Vaccine-based immunotherapy and immune checkpoint targeted therapy may only be effective for some subpopulations expressing specific genes (6, 7). The immunosuppressive TME dramatically reduces the effectiveness of immunotherapy (8). Thus, a thorough understanding of the unique immunological status in heterogeneous glioma TME will be essential for the clinical application of glioma immunotherapy.

Integrin subunit alpha 5 (ITGA5) encodes a protein belonging to the integrin alpha chain family, which plays a vital function in cell surface adhesion and signaling. ITGA5 has a deep connection with tumor invasion, tumor progression, and chemotherapy resistance (9, 10). ITGA5 was shown to be of prognostic use in non-small cell lung cancer (11) and breast cancer bone metastasis (12). The ITGA5 monoclonal antibody M200 could effectively reduce bone metastasis and blunt cancer-associated bone destruction (12). ITGA5 was regulated in glioma cells and mediated glioma cell dispersion and invasion by cell–matrix and cell–cell interactions (13). Meanwhile, ITGA5 orchestrated the process of proliferation inhibition induced by tocopherols of glioma cells (14). Therefore, ITGA5 is an important gene modifier in oncogenesis and tumor development. More importantly, emerging evidence also certified that ITGA5 could function as a determinant of immune cell infiltration and promising prognostic biomarkers in gastrointestinal tumors (15). Liu et al. also constructed an ITGA5-comprising risk model based on eight genes, which was strongly related to the immune infiltration patterns in breast cancer (16). These results supported the possibility that ITGA5 modulated the immune infiltration characteristics and showed great prognostic value in tumor patients.

Overall, given the pivotal role of ITGA5 in malignancies, we hypothesized that a comprehensive elucidation of the ITGA5-related multi-omics landscape, especially TME contexture, may favor the deeper understanding of the gliomas pathogenesis and progression. In this study, we comprehensively investigated the ITGA5 expression patterns in gliomas based on genomic and transcriptional profiles with complete clinical annotations. To get more insights, immunological characteristics, functional annotation, chemotherapeutic response prediction, and overall survival (OS) were analyzed to interpret the correlation between ITGA5 and glioma tumor immune microenvironment. Collectively, our comprehensive analyses deciphered the prognostic, immunological, and therapeutic value of ITGA5 in glioma management, thus providing a target for individual and precise therapy for combating gliomas.

## Materials and Methods

### Glioma Datasets and Preprocessing

The pan-cancer related data and the corresponding clinical information were downloaded and collected from The Cancer Genome Atlas (TCGA; https://xenabrowser.net/). Normal sample data were collected from the Genotype-Tissue Expression Project (GTEx; https://www.gtexportal.org). The glioma genomic information together with complete clinicopathological annotations was obtained from the TCGA, the Chinese Glioma Genome Atlas (CGGA; http://www.cgga.org.cn/), and the Gene Expression Omnibus (GEO; https://www.ncbi.nlm.nih.gov/geo/). In total, 9,807 pan-cancer patients containing 33 cancer types, and 8,295 normal samples containing 31 kinds of normal tissues, were involved. In addition, the transcriptional profiles of 3,047 glioma patients were acquired from 11 cohorts containing greater than 50 patients and samples with inadequate OS information were excluded. Specific information of the patients and the corresponding platforms are presented in **Table S1**.

Affymetrix and Agilent platforms were utilized for generating the raw data derived from the GEO database. The robust multichip average (RMA) algorithm was utilized to achieve background correction and normalization. The TCGA and the CGGA data portals provided the RNA-sequencing data. The fragments per kilobase million (FPKM) values were transformed into transcripts per kilobase million (TPM) values that possessed similar signal intensity with the RMA-processed values (17).

### Genomic Alteration

The somatic mutations and somatic copy number variation (CNV) profiles were gathered from the TCGA datasets. The Genomic Identification of Significant Targets in Cancer (GISTIC) analysis was performed to evaluate the genomic features. The CNV landscape based on ITGA5 levels and the copy number gains or losses at the amplified or deleted peaks were assessed by GISTIC 2.0 analysis (https://gatk.broadinstitute.org) (18).

### Evaluation of the Immunological Characteristics of the TME

The intratumoral immune cell abundance, stromal cell infiltration levels, and tumor purity were estimated by The Estimation of Stromal and Immune cells in Malignant Tumor tissues using Expression (ESTIMATE) algorithm, and reflected by immune score, stromal score, and estimate score separately (19). The Tumor Immune Estimation Resource2.0 (TIMER2.0; http://timer.cistrome.org/) web server (20) was utilized for comprehensively analyzing the level of immune infiltrating cells in gliomas. The relative fraction of 10 types of immune cells in the tumor was estimated using the MCPcounter algorithm (21). The infiltration levels of 28 immune cells were presented by the enrichment scores based on corresponding signatures. The enrichment scores were calculated by the single sample gene set enrichment analysis (ssGSEA) implemented using the R gene set variation analysis (GSVA) package (22). The cancer immunity cycle reflected the anticancer immune response and comprises seven steps. The activities of these steps determined the fate of the tumor cells, which were evaluated the activities of these steps using the ssGSEA (23). The immune checkpoints, related to seven different immune processes, were retrieved from two prior literature (24, 25). The responses to immune checkpoint blockade immunotherapy in gliomas were extrapolated by the Tumor Immune Dysfunction and Exclusion (TIDE) algorithm (26). The responses to anti-PD1 and anti-CTLA4 therapies in gliomas were evaluated by the submap algorithm.

### Functional Annotation

All gene sets derived from the Gene Ontology (GO) and the Kyoto Encyclopedia of Genes and Genomes (KEGG) were downloaded from the MSigDB database (27). Metabolism-relevant gene signatures were introduced previous study (28). Gene set enrichment analysis (GSEA) and GSVA based on ITGA5 transcriptional abundance were implemented by the clusterProfiler R package and GSVA R package (29).

### Drug Response Prediction

The pharmacogenomic data sourced from the Genomics of Drug Sensitivity in Cancer (GDSC, https://www.cancerrxgene.org/) was used from predicting the drug susceptibility of included cases. The drug responses were performed as half-maximal inhibitory concentration (IC50) calculated by the pRRophetic R package.

### Statistical Analysis

The survival of different groups was ascertained by Kaplan–Meier curves (KM curves) and compared using the log-rank test. We used the uni-Cox and multi-Cox regression analyses to test the independence of the prognostic factors and evaluate the hazard ratio. Pearson correlation and distance correlation analyses were used to calculate correlation coefficients. Contingency tables were analyzed by Fisher test. Based on the ITGA5 expression, patients were grouped as a high- or low-group. Most data visualizations were completed with the R ggplot2. OncoPrint delineating the mutation landscape was generated by the R maftools (30). All KM curves were implemented using the R survminer. Heatmaps were visualized based on complexHeatmap (31). All statistical analyses were conducted with R sv3.6.3 (https://www.r-project.org). P <0.05 was considered statistically significant.

### Multiple Immunofluorescence (IF)

The gliomas chip was baked at 60°C for 60 min for deparaffinization, then the antigen was retrieved by EDTA retrieval buffer and blocked in 3% BSA. Next, the samples were permeabilized with 0.1% Triton X-100. The gliomas tissue microarray was incubated with the primary antibodies of ITGA5 (10569-1-AP, Rabbit, 1:200, Proteintech, China), CD68 (GB113150, Rabbit, 1:3,000, Servicebio, China), and CD163 (16646-1-AP, Rabbit, 1:3,000, Proteintech, China) separately, then followed by the incubation with secondary antibodies (GB23301, GB23303, Servicebio, China) and tyramide signal amplification (TSA) [FITC-TSA, CY3-TSA, and CY5-TSA (Servicebio, China)]. The antigen repair was applied repeatedly between the intervals of each dye. Subsequently, the microarray was incubated with 4’,6-Diamidino2-phenylindole dihydrochloride (DAPI). Microscopy detection was performed by the Pannoramic Scanner (3D HISTECH, Hungary).

## Results

### ITGA5 Expression Was Positively Related to Aggressive Clinicopathological and Molecular Features in Gliomas

**Figure 1:**
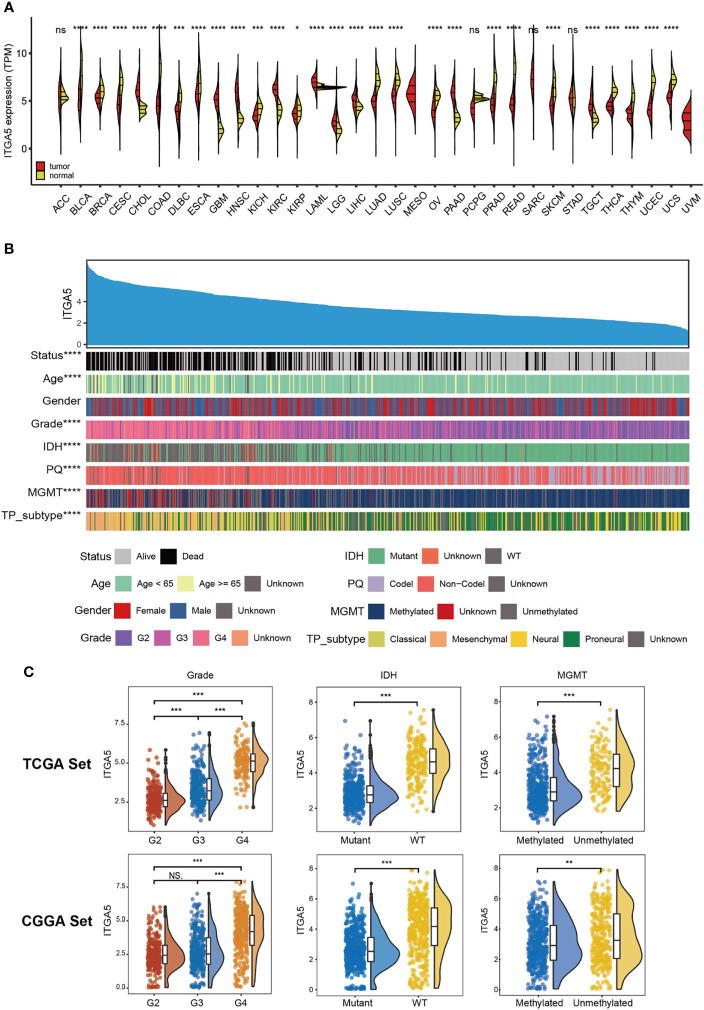
The clinical and molecular characteristics in associations with ITGA5 expression. **(A)** ITGA5 levels among pan-cancer samples grouped by cancer and normal status from TCGA and GTEx. **(B)** An overview of the association between known clinical and molecular features in TCGA, namely, status, age, gender, grade, IDH mutation, 1p/19q codeletion, MGMT methylation, and TP subtype. **(C)** The expression levels of ITGA5 in different WHO grades, IDH states and MGMT methylational states from the TCGA and the CGGA datasets. NS, not statistically significant, *P < 0.05, **P < 0.01, ***P < 0.001, ****P < 0.0001.

Based on the TCGA and the GTEx, we firstly examined the ITGA5 transcriptional abundance in 33 tumor types. The malignancy-related overexpressed patterns of ITGA5 were identified in cholangiocarcinoma (CHOL), head and neck squamous cell carcinoma (HNSC), kidney renal clear cell carcinoma (KIRC), acute myeloid leukemia (LAML), liver hepatocellular carcinoma (LIHC), pancreatic adenocarcinoma (PAAD), testicular germ cell tumors (TGCT), especially, brain lower-grade glioma (LGG) and glioblastoma multiforme (GBM) **(** **Figure 1A** **)**. In gliomas, the ITGA5 transcriptional levels increased with more adverse clinicopathological characteristics, namely, greater age at diagnosis, 1p/19q non-codeletion, higher grade, wild-type isocitrate dehydrogenase (IDH) status, unmethylated MGMT status, and mesenchymal subtype **(** **Figure 1B** **)**. Moreover, the ITGA5 amplification in gliomas with high-grade or invasive molecular signatures was validated both in the TCGA and the CGGA cohorts **(** **Figure 1C** **)**. These results prompted that the ITGA5 expression was enhanced with the progression of glioma.

### Elevated ITGA5 Levels Were Associated With Poor Survival of Glioma Patients

**Figure 2:**
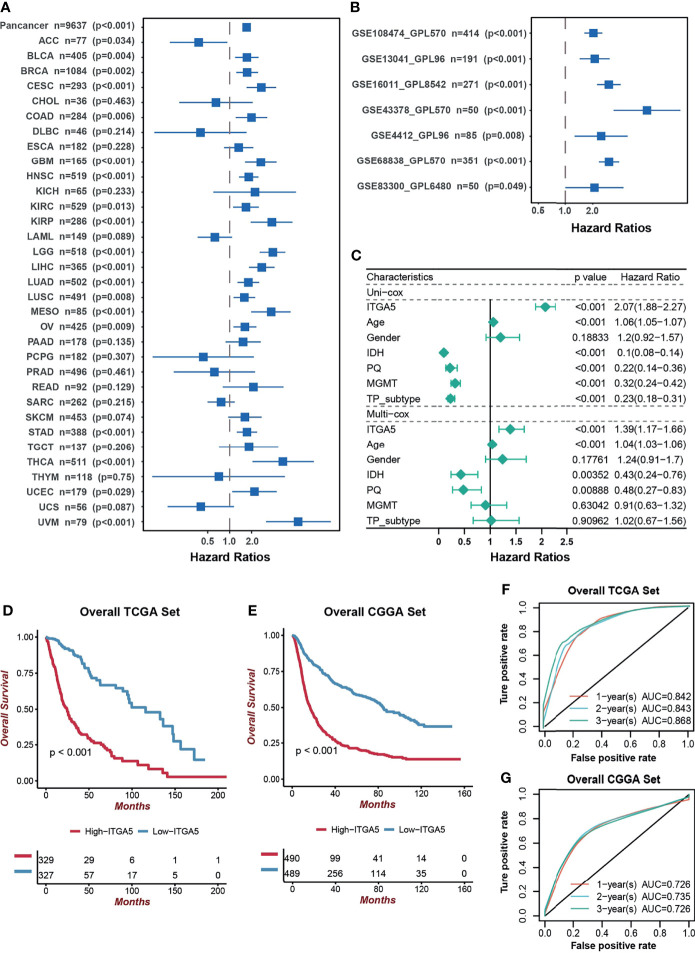
The prognostic potential of ITGA5. **(A)** Univariate cox analysis of ITGA5 for overall survival of patients in the TCGA pan-cancer cohorts. **(B)** Univariate Cox analysis for overall survival of patients with gliomas based on the GEO datasets. **(C)** The forest plot of univariate and multivariate cox proportional hazard ratios for ITGA5 based on the TCGA dataset. **(D)** Kaplan–Meier curves for high and low ITGA5 level groups in the TCGA. **(E)** Kaplan–Meier curves for high and low ITGA5 level groups in the CGGA. **(F)** The time-dependent receiver operating characteristic curve of ITGA5 from the TCGA. **(G)** The time-dependent receiver operating characteristic curve of ITGA5 from the CGGA.

Next, we focused on the prognostic value of ITGA5 in a pan-cancer setting. The result of the Kaplan–Meier analysis demonstrated that ITGA5 was confirmed as a significant protective factor only in adrenocortical carcinoma (ACC). However, ITGA5 was identified as a mortality risk factor in a wide spectrum of cancer types, namely, bladder urothelial carcinoma (BLCA), breast invasive carcinoma (BRCA), cervical squamous cell carcinoma and endocervical adenocarcinoma (CESC), colon adenocarcinoma (COAD), HNSC, KIRC, kidney renal papillary cell carcinoma (KIRP), LIHC, lung adenocarcinoma (LUAD), lung squamous cell carcinoma (LUSC), mesothelioma (MESO), ovarian serous cystadenocarcinoma (OV), stomach adenocarcinoma (STAD), thyroid carcinoma (THCA), uterine corpus endometrial carcinoma (UCEC), uveal melanoma (UVM), notably, LGG and GBM **(** **Figure 2A** **)**. As expected, the adverse prognostic impacts of ITGA5 were obtained from 7 independent glioma sets from the GEO database **(** **Figure 2B** **)**. Additionally, the uni-Cox and multi-Cox analyses indicated that ITGA5 was the independent indicator for the mortality in glioma patients **(** **Figure 2C** **)**. Subsequently, the TCGA-based and the CGGA-based KM curves more firmly demonstrate the severe survival detriment in glioma patients with high ITGA5 expression **(** **Figures 2D, E** **)**. The conspicuously impaired survival in high-ITGA5 groups was observed in 7 independent glioma sets from the GEO database as well **(** **Figure S1** **)**. Moreover, the time-dependent receiver operating characteristic (ROC) curves of ITGA5 exhibited high sensitivity and specificity, which were demonstrated by the 1-, 2-, and 3-year all area under the curves (AUC) were greater than 0.842 in the TCGA gliomas dataset, and greater than 0.726 in the CGGA gliomas dataset, respectively **(** **Figures 2F, G** **)**. In conclusion, the higher ITGA5 abundance was closely associated with a higher mortality hazard in glioma patients. Therefore, ITGA5 could be exploited as a powerful indicator of clinical outcomes in gliomas.

### ITGA5 Was Involved in the Oncogenic Process and Immune Regulation in Gliomas

**Figure 3:**
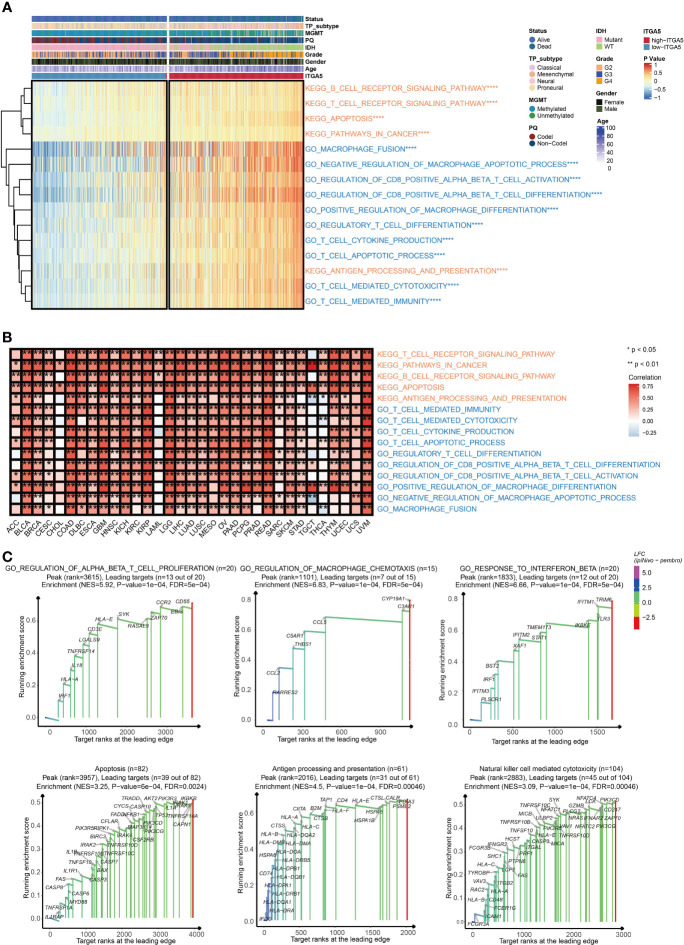
The functional annotation based on ITGA5 expression. **(A)** The heatmap for gene set variation analysis of the ITGA5 from the TCGA. *P < 0.05, **P < 0.01, ***P < 0.001, ****P < 0.0001. **(B)** The heatmap showing the relationship between the above pathways and ITGA5 in the TCGA pan-cancer cohorts. **(C)** GSEA plots for several signaling pathways positively regulated by ITGA5.

To explore the potential pathological function of ITGA5, the GSVA analysis was performed in the TCGA gliomas cohort. The results revealed that ITGA5 contributed to the aberrant activities of several pivotal pro-oncogenic behaviors, such as focal adhesion, glycolysis gluconeogenesis, Janus kinase (JAK)/signal transducer, and activator of transcription (STAT) signaling pathway, P53 signaling pathway, and the proteasome. Besides, the amplification of ITGA5 also potentiated a plethora of immune-related processes, namely, interleukin-4-(IL-4) mediated signaling pathway, macrophage fusion, negative regulation of macrophage apoptotic process, positive regulation of macrophage differentiation, and positive regulation of regulatory T cell differentiation **(** **Figure 3A** **)**. It was worth emphasizing that the modulation of ITGA5 of these pathophysiological activities, especially focal adhesion, was highly prevalent across multiple cancers **(** **Figure 3B** **)**. The GSEA analysis reaffirmed the crucial role of ITGA5 in carcinogenesis and shaping tumor immune microenvironment, nicely exemplified by priming the regulation of T cell proliferation, regulation of macrophage chemotaxis, response to interferon-beta, apoptosis, antigen processing and presentation, natural killer cell-mediated cytotoxicity **(** **Figure 3C** **)**. Overall, the high expression of ITGA5 supplied a fertile niche favoring glioma progression by activating oncological signaling pathways and immune reprogramming.

### ITGA5 Expression Was Relevant to Distinct Genomic Alterations

**Figure 4:**
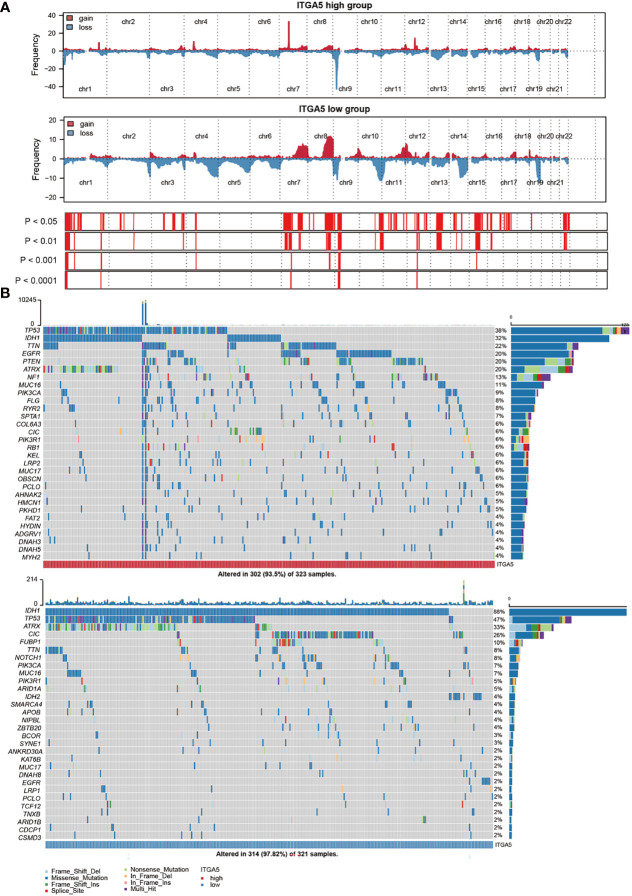
ITGA5-associated genomic alterations in gliomas samples. **(A)** Main copy-number changes in gliomas with high and low ITGA5 expression. **(B)** Somatic mutations detected in gliomas with high and low ITGA5 expression.

Genomic instability contributes to multiple steps of the gliomas progressive trajectory. To explore the underlying genetic regulation role of ITAG5, the CNV and somatic mutation analyses were taken in the TCGA glioma dataset to present the ITGA5-based genomic landscapes. The global CNV profile revealed that the amplification in the high-ITGA5 group was concentrated on chr 7 and chr 12, specifically, 7p11.2 and 12q14.1, and the deletion concentrated on chr9, specifically, 9p21.2 and 9p21.3. In the low-ITGA5 group, it was indicated that chr 12 gain, chr 2 loss, chr 10 loss, and chr 11 loss, and the amplification was mainly found on 12p13.32, the deletion was mainly focused on 2q37.1, 2q37.3,10q26.3, and 11p15.5 **(** **Figure 4A**, **Figure S2A** **)**. The detailed amplificated or deleted CNV oncoplots are presented in **Figure S2B**. The global views of mutational distribution showed that cellular tumor antigen p53 (TP53) mutation was enriched in both the high-ITGA5 group and the low-ITGA5 group (38 and 47%, respectively) **(** **Figure 4B** **)**. Besides, isocitrate dehydrogenase [NADP] cytoplasmic (IDH1) mutation was generally presented in the low-ITGA5 group as 88% and was partially observed in the high-ITGA5 groups as 32%. The three followed most frequently mutated genes were titin (TTN) (22%), epidermal growth factor receptor (EGFR) (20%), and phosphatase and tensin homolog (PTEN) (20%) in the high-ITGA5 group, and was alpha-thalassemia/mental retardation syndrome x-linked chromatin remodeler (ATRX) (33%), capicua transcriptional repressor (CIC) (26%) and far upstream element binding protein (FUBP1) (10%) in the low-ITGA5 group **(** **Figure 4B** **)**.

Furthermore, the ratio of mutation frequencies between high-ITGA5 and low-ITGA5 groups was performed by Fisher’s exact test and ranked by increasing p-value. The high-ITGA5 group beard extremely lower IDH1 and CIC mutation load, higher PTEN, EGFR, and NF1 mutation load than the low-ITGA5 group **(** **Figure S3A** **)**. Additionally, the co-concurrent or mutually exclusive mutations of the 25 most frequently mutated genes are shown in **Figure S3B**. The high-ITGA5 groups showed markedly more numerous co-concurrent genetic alterations than the low-ITGA5 group. The EGFR mutation frequently cooccurred with collagen type VI alpha 3 chain (COL6A3) mutation in the high-ITGA5 group. Other intimate mutation sites included obscurin (OBSCN) and COL6A3, IDH1 and TP53, ATRX, and TP53. In the low-ITGA5 group, the common co-mutation included NOTCH1 and CIC, IDH1 and ATRX, and so on. Meanwhile, some intensive mutually exclusive pairs of gene alteration were identified, such as IDH1-PTEN and TP53-EGFR in the high-ITGA5 group, and IDH1-EGFR and IDH1-IDH2 in the low-ITGA5 group **(** **Figure S3B** **)**.

### ITGA5 Was Correlated With Tumor Immune Microenvironment in Gliomas

**Figure 5:**
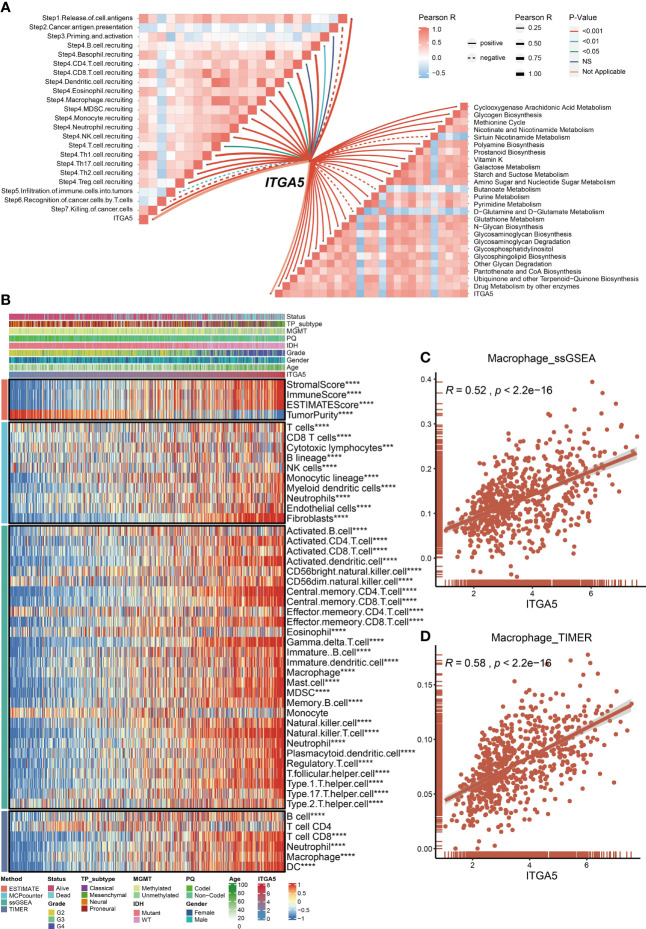
Roles of ITGA5 in immune and metabolism phenotypes in the TCGA cohort. **(A)** Correlations between ITGA5 and enrichment scores of metabolism-relevant pathways together with the steps of the cancer immune cascades. **(B)** Heatmap visualized the abundance of infiltrating immune cell populations with different ITGA5 levels, based on the ESTIMATE, the MCPcounter, the ssGSEA, and the TIMER algorithms. *P < 0.05, **P < 0.01, ***P < 0.001, ****P < 0.0001. **(C)** The corrplot of the correlation between ITGA5 expression and macrophage cell level of the ssGSEA. **(D)** The corrplot of the correlation between ITGA5 expression and macrophage cell level of the TIMER.

Metabolism has been well-established as a determinant in the viability and efficacy of immune cells (32). Then, the physiopathological impact of ITGA5 in the metabolic programming and cancer immunity cycle progressions was evaluated by GSVA analysis. Based on the TCGA dataset, ITGA5 was positively correlated with glycogen biosynthesis, cyclooxygenase arachidonic acid metabolism, and drug metabolism by other enzymes **(** **Figure 5A** **)**. Another noteworthy observation was that ITGA5 exerted a pleiotropic effect in most steps of the immune cascade **(** **Figure 5A** **)**. Considering that immune cells were the essential constituents of the TME, the immune infiltration characteristics were identified by the ESTIMATE, the MCP counter, the ssGSEA, and the TIMER algorithms of the TCGA datasets **(** **Figure 5B** **)**. Notably, the ITGA5 expression showed a particularly positive correlation with the stromal score, immune score, ESTIMATE score, and tumor purity. Besides, ITGA5 expression was associated with the high intratumoral infiltration of various immune cells, namely, macrophages, natural killer cells, CD8^+^ T cells, and regulatory T cells (Tregs). Then, we further explored the relationship between macrophage infiltration and ITGA5 from the ssGSEA and the TIMER **(** **Figures 5C, D** **)**.

### High-ITGA5 Group in Gliomas Exhibited Greater M2 Macrophage Infiltration

**Figure 6:**
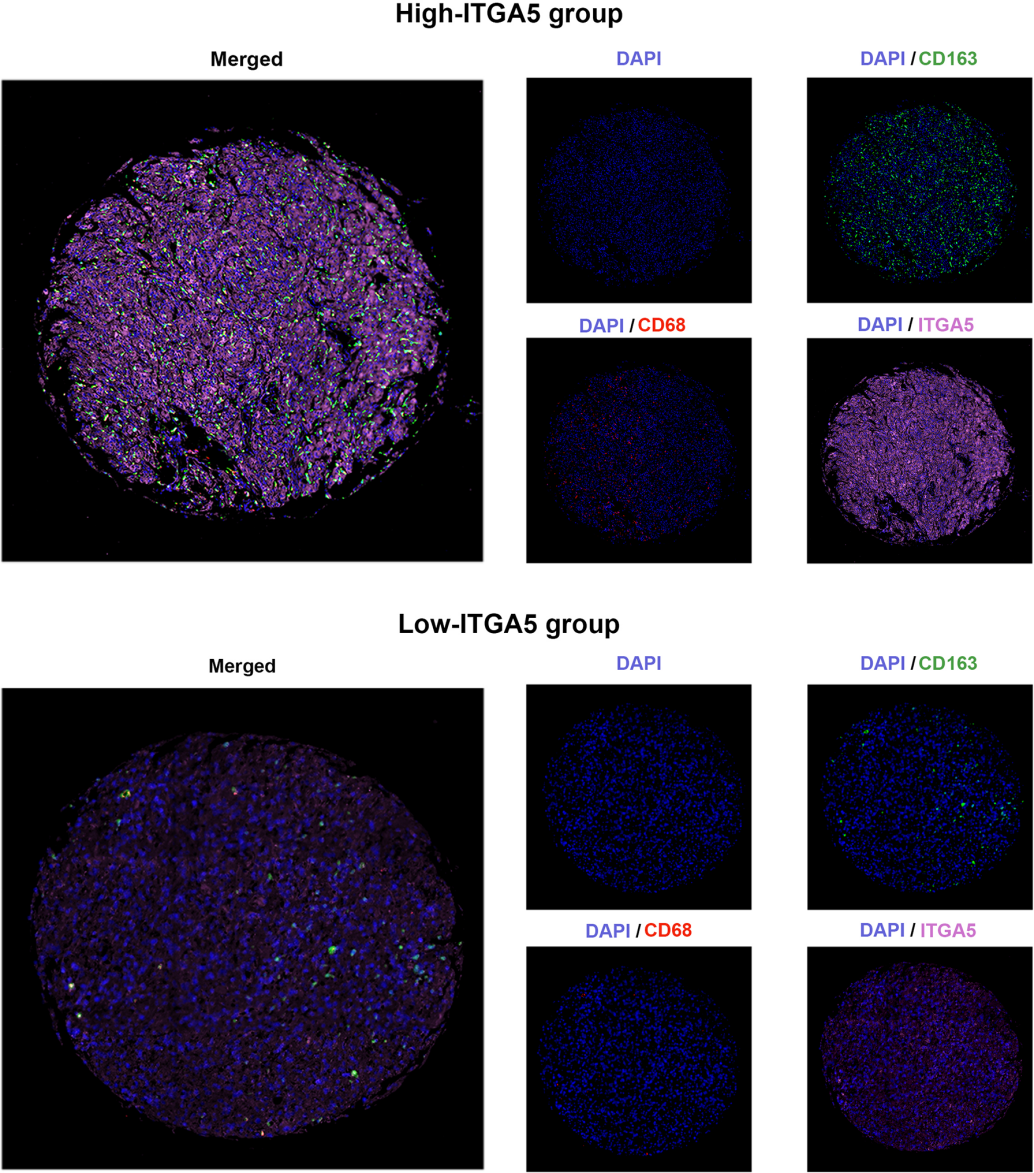
The multiple immunofluorescence staining of ITGA5, CD68, and CD163 in gliomas.

To confirm the positive correlations between ITGA5 and macrophages identified from transcriptomic analyses, the multiple IF was performed on the human glioma tissue microassay. Staining for CD68 (the pan-macrophage marker) revealed that there was more diffuse intra-tumoral macrophage infiltration in the high-ITGA5 group than in the low-ITGA5 group **(** **Figure 6** **)**. Of more concern was that the high-ITGA5 group displayed a higher M2-macrophage abundance, which was prompted by CD163 (the M2-macrophage marker) fluorescence distribution **(** **Figure 6** **)**.

### The Potential Immunotherapeutic and Chemotherapeutic Targets in Glioma Patients With High ITGA5 Expression

**Figure 7:**
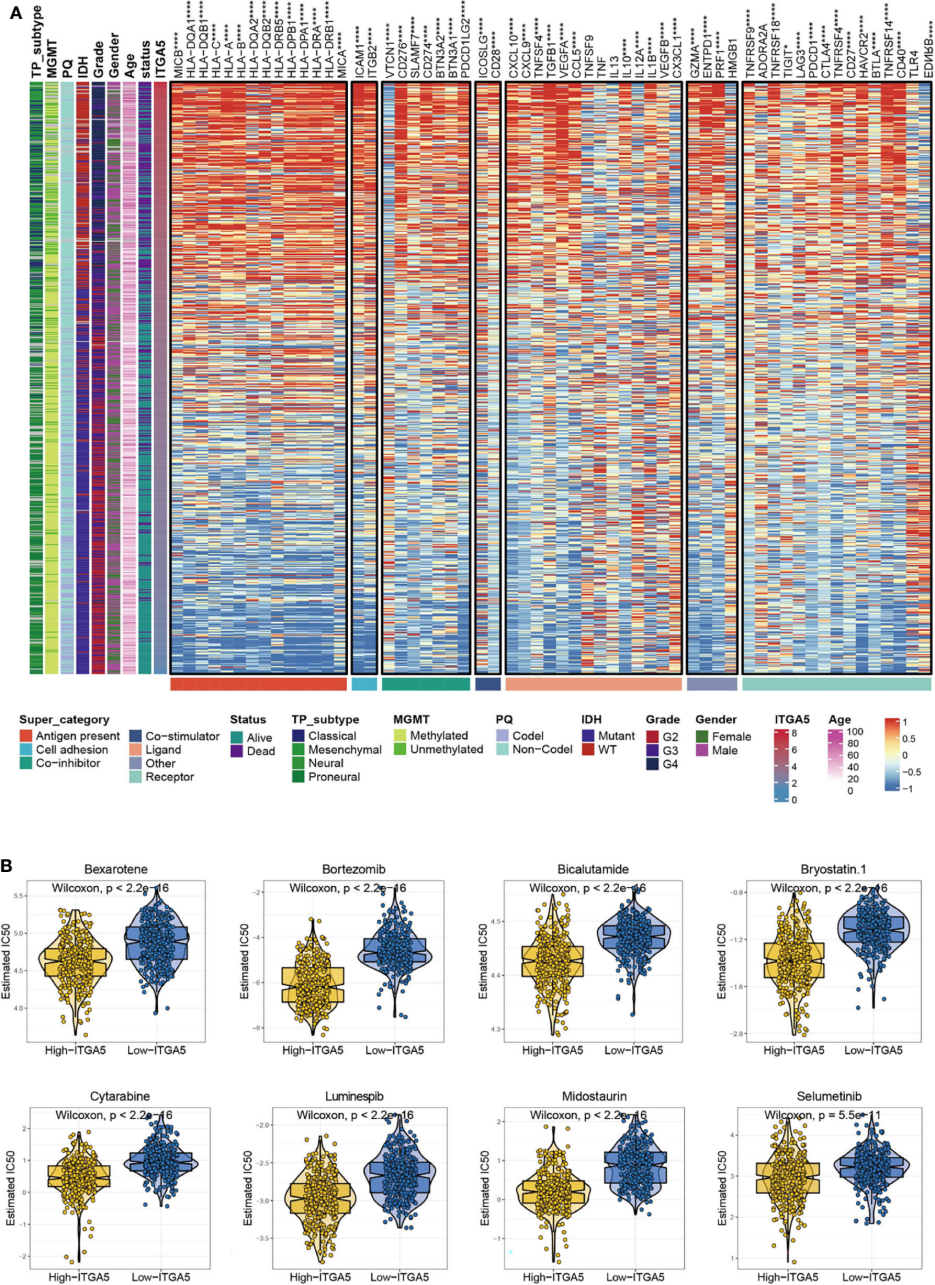
The potential ITGA5-involved immune checkpoints treatment and chemotherapeutic targets in gliomas. **(A)** Correlations between ITGA5 and seven types of immune checkpoints levels in gliomas. *P <0.05, **P <0.01, ***P <0.001, ****P <0.0001. **(B)** The box plots of the estimated IC50 for several chemotherapeutic drugs among high-ITGA5 and low-ITGA5 groups.

Given that immune checkpoints are the underpinnings of immune therapy, the global views of immune-checkpoints expressions would be of great clinical interest. Then the correlation analysis was performed between ITGA5 and multiple putative immune checkpoint molecules involving antigen presentation, cell adhesion, co-inhibition or co-stimulation, and ligand-receptor interaction. The results exhibited an intimate connection between ITGA5 and most immune checkpoint molecules in gliomas, especially in programmed cell death 1 (PD-1), programmed cell death 1 ligand 1 (PD-L1), and cytotoxic T-lymphocyte associated protein 4 (CTLA-4) **(** **Figure 7A** **)**. Besides, additional validation of the intimate connection of ITGA5 and immune checkpoints was performed in the pan-cancer set **(** **Figure S4** **)**. These data collectively suggested that ITGA5 inhibition may work synergistically with existing immunotherapy strategies and enhance anti-glioma activity. We further explored the relationship between ITGA5 and some immune checkpoints, such as PDCD1 (PD-1), CD274 (PD-L1), and PDCD1LG2 (PD-L2) in gliomas based on the TCGA dataset **(** **Figure S5C** **)**.

To further evaluate the sensitivity of ITGA5 to immunotherapy, we applied the TIDE and the submap algorithms to glioma patients in the TCGA. By TIDE, we found that patients with high ITGA5 showed better immunotherapy response compared to patients with low ITGA5 (**Figure S5A**). The results of the submap showed that the high- and low-ITGA5 groups had different responses to immunotherapy in that the high-ITGA5 group had a significant response to anti-PD-1 immunotherapy in gliomas based on the TCGA (**Figure S5B**).

There has been a regrettable case that conventional chemotherapy with temozolomide provides modest benefit for the survival of gliomas patients, which may impute to the impaired drug permeability owing to the BBB, the particular immune contexture in the central nervous system, or the extreme heterogeneity of gliomas. Thus, the GDSC database was adopted to assess the drug response of high-ITGA5 and low-ITGA5 glioma groups, and the predictive accuracy was measured by 10-fold cross-validation. The results demonstrated that high-ITGA5 groups exhibited significantly lower IC50 of Bexarotene, Bicalutamide, Bortezomib, Bryostatin-1, Cytarabine, Luminespib, Midostaurin, Selumetinib than the low-ITGA5 group **(** **Figure 7B** **)**. The therapeutic efficacy of the above-nominated pharmacological methods deserved further investigation.

## Discussion

Glioma has a lethal ending with limited treatment methods, the highest grade of which has an OS of about 15 months (33). The heterogeneity and complicated immunosuppressive TME are the main obstacles for glioma patients with optimal outcomes. The systematical application of novel excavated biomarkers will be conductive to glioma management in combination with conventional diagnostic and therapeutic strategies. In the present study, by comprehensive bioinformatic analysis, we identified that ITGA5 was positively related to aggressive clinicopathological and molecular features in gliomas. Elevated ITGA5 level was tightly linked to the survival detriment of gliomas patients. Moreover, ITGA5 was relevant to distinct genomic alterations and tumor immune microenvironment in gliomas. ITGA5 could be a therapeutic indicator of checkpoint inhibitors as well. Finally, the glioma patients with higher expression of ITGA5 may present better outcomes of chemotherapy treatment, namely, bexarotene, bicalutamide, and bortezomib, which provided more therapeutic choices in the future.

ITGA5 is also known as interferon α5 belonging to the integrin alpha chain family, which plays a pivotal role in cell surface adhesion. Biologically, interferon α subunits have a small cytoplasmic tail, a relatively large extracellular domain, and a transmembrane domain. In pathological settings, integrin demonstrated a remarkable oncogenic potency in tumor progression. The extracellular domain acts as the cell receptor for growth factors and adhesion proteins from the TME (34). The cytoplasmic tail is a link to the cell cytoskeleton and cellular signaling cascade like focal adhesion kinase (FAK)-Src signaling (34). The FAK-Src signaling regulated by integrin allows downstream activation of several signaling pathways, namely, the Mitogen-Activated Protein Kinase (MAPK)/Extracellular Regulated Kinase (Erk) signaling pathway, phosphatidylinositol 3-kinase (PI3-K)/Protein kinase B (Akt) signaling pathway, Stress-Activated MAP Kinases (SAPKs) signaling, and c-Jun N-terminal kinase (JNK) activation (35). The activation of PI3K/Akt is the key regulator of induction of tumor metastasis through the engagement of extracellular matrix ligand (36). MAPK/Erk pathways are highly related to tumor progression, survival, and proliferation (37). In our results, ITGA5 was found to mediate lots of different oncological and immune pathways such as glycolysis gluconeogenesis, JAK/STAT signaling pathway, P53 signaling pathway, antigen processing pathway, and immune cell activation pathway. The dysregulated metabolism of the tumor and immune cells in TME is recognized as a key contributor to tumor progression. Our results prompted that ITGA5 may perturb the biochemical homeostasis in TME by not only oncogenic pathway but also immune and metabolic reprogramming, which was partially consistent with the previous oncological studies.

Genomic alteration is one of the fundamental factors for diagnosis, classification, treatment, and prognosis in gliomas. The typical biomarkers of genomic alteration are routinely tested in clinical practice, namely, IDH mutant, MGMT promoter methylation, PTEN mutation, ATRX mutation, and TERT promoter mutation (38). Surprisingly, although patients underwent the systematic diagnosis and treatment depending on the WHO classification, the prognosis varies differently, strongly implying that the current diagnosis system is not fully appropriate for the survival predictions or the reflection of glioma heterogeneity. Chen reported a phenomenon that the ITGA5 downregulation could inhibit the glioma proliferation, which could be rescued by circPTN (39). Moreover, Kita also confirmed that ITGA5, which was upregulated by Ets-1, could be secreted by glioma cells to boost tumor migration and invasion, finally contributing to glioma malignancy (40). Consistently, our results also revealed that ITGA5 might be an oncogenic factor, presuming that the high ITGA5 expression possibly indicated a low survival rate in glioma patients. In the other aspect, our genomic alterations analysis results showed that ITGA5 was associated with poor mutation, namely, P53 mutation, ATRX mutation, and PTEN mutation. Especially, the high expression of ITGA5 had relatively lower IDH mutations. IDH mutation is a classic prognostic indicator in judging glioma, and there is a certain negative correlation between ITGA5 expression level and IDH mutation level.

A glioma has a different immunosuppressive “cold” tumor environment affecting the effectiveness of immunotherapy (41). TAMs are the most abundant infiltration immune cells in brain cancer validated from high-dimensional single-cell profiling and other methods (42). TAMs contribute to tumor immunosuppressive environments and potentially mediate tumor-induced polarization states, promoting tumor malignancy, and therapeutic resistance to irradiation (43). In our results, from the ssGSEA and the TIMER analyses, ITGA5 was positively correlated with TAMs, possibly resulting in a worse survival rate. Furthermore, it also found that upregulated ITGA5 significantly increased the quantities of multiple immune cells, namely, dendritic cells, natural killer cells, CD8^+^ T cells, neutrophils, activated CD4^+^ T cells, central memory T cells, Tregs, and myeloid-derived suppressor cells. Among these cells, immunosuppressive cells such as Tregs and myeloid-derived suppressor cells were increased more significantly with the increase of ITGA5 expression, suggesting that ITGA5 was essential in the formation of glioma immunosuppressive microenvironment (44). It is well-documented that the immune checkpoint targeted therapy in glioma patients remains challenging because of the immunosuppressive state (6). Besides, we also proved that high-ITGA5 gliomas were accompanied by high immunotherapy biomarker expression, namely, PD-1, PD-L1, CTLA-4, and VEGF, thus showing an ITGA5-targeted therapeutic potential to be synergistic with immunotherapy. Therefore, our results presented that the high ITGA5 expression patients seem to have a relative “hot” immune environment that might be beneficial to immune therapy.

Up to now, there are emerging studies devoted to developing novel and effective strategies to address the deficiencies of anti-glioma treatment. EGFR has been the target of treatment in glioma patients (45) both in a highly selective pan-human EGFR inhibitor or immunotherapies (46). Interestingly, ITGA5 depletion delayed gefitinib-mediated EGFR endocytosis, which makes EGFR target therapy more sensitive (47). In addition, the inhibitor of ITGA5 attenuates glioma growth (48) and cell dispersion (13). In our results, we found that patients with high expression of ITGA5 had low IC50 of Bexarotene, Bicalutamide, Bortezomib, Bryostatin-1, Cytarabine, Luminespib, Midostaurin, and Selumetinib. This result emphasized that the high ITGA5 expression was a robust and reliable indicator for the therapeutic sensitivity of these potential molecular drugs. The biosafety and biocompatibility of these drugs in clinical trials have been successfully experimentally validated. Among them, it was noted that some drugs, represented by Bexarotene, Bryostatin-1, Cytarabine, and Pazopanib, possess the excellent capability of crossing BBB to locally exert a targeted effect. This might provide a pivotal clue for guiding glioma drug treatment.

However, there are still some limitations. In the first place, since this study data analysis are mainly from the open-access online databases, additional external verification of clinical datasets would be beneficial. Secondly, because this is a retrospective study, and prospective clinical studies in large-cohorts should be applied to verify in the future. Thirdly, there is a relatively poor investigation of the mechanism of ITGA5 and its role in interfering with the immune system. Thus more comprehensive studies are needed to further interpret the role of ITGA5 in gliomas immunology.

## Conclusion

In summary, our findings demonstrated that ITGA5 is upregulated in glioma and differs in multiple molecular phenotypic gliomas, acting as a potential biomarker for predicting glioma prognosis. It is particularly noteworthy that ITGA5 is involved in remolding glioma immune infiltration and TME that is closely related to immunotherapy. High expression of ITGA5 may benefit immune checkpoint targeted therapy and chemotherapy. Further studies exploring the role and mechanism of ITGA5 will endow great potential for the development of diagnostic and anti-glioma therapeutic strategies.

## Data Availability Statement

The datasets presented in this study can be found in online repositories. The names of the repository/repositories and accession number(s) can be found in the article/**Supplementary Material**.

## Ethics Statement

All procedures performed in studies involving human participants were in accordance with the ethical standards of the institutional and/or national research committee and with the 1964 Helsinki declaration and its later amendments or comparable ethical standards.

## Author Contributions

Shu-YL and NZ contributed equally as co-first authors. Shu-YL and NZ conceived and designed the study. Shi-YL and HZ. drafted the manuscript. J-JL. did the statistical analysis, supervised by Y-WQ, QZ, and X-RL. All authors listed have made a substantial, direct, and intellectual contribution to the work and approved it for publication.

## Funding

This work was supported by the National Natural Science Foundation of China [Grant Nos. 81802676] and the Wuhan Youth Cadre Project (2017zqnlxr01 and 2017zqnlxr02).

## Conflict of Interest

The authors declare that the research was conducted in the absence of any commercial or financial relationships that could be construed as a potential conflict of interest.

## Publisher’s Note

All claims expressed in this article are solely those of the authors and do not necessarily represent those of their affiliated organizations, or those of the publisher, the editors and the reviewers. Any product that may be evaluated in this article, or claim that may be made by its manufacturer, is not guaranteed or endorsed by the publisher.

## Supplementary Material

Supplementary Figure 1 The Kaplan–Meier curves of ITGA5 in 7 independent GEO datasets.

Supplementary Figure 2 The genomic alterations in high-ITGA5 and low-ITGA5 groups. **(A)** Amplification and deletions in gliomas with high and low ITGA5 expression. **(B)** The detailed amplification and deletion of chromosome copy number variation in gliomas with high and low ITGA5 expression.

Supplementary Figure 3 The somatic mutations in high-ITGA5 and low-ITGA5 groups. **(A)** The forest plot illustrates the difference in mutation patterns between high-ITGA5 and low-ITGA5 groups. **(B)** The heatmap presents the somatic interaction in gliomas with high and low ITGA5 levels.

Supplementary Figure 4 The correlation between ITGA5 expression and recognized immune checkpoints in pan-cancer.

Supplementary Figure 5 The evaluation of the sensitivity of ITGA5 to immunotherapy. **(A)** TIDE predicting the immunotherapy responses related to ITGA5 in gliomas. **(B)** Submap analysis of ITGA5 expression levels in gliomas. **(C)** The corrplot of the correlation between ITGA5 expression and immune checkpoints.

Supplementary Table 1 Specific information of the patients and the corresponding platforms.

## Glossary

**Table:**

MGMT	O-6-methylguanine-DNA methyltransferase
TME	tumor microenvironment
TAMs	tumor-associated macrophages
BBB	blood–brain barrier
ITGA5	integrin subunit alpha 5
TCGA	The Cancer Genome Atlas
GTEx	Genotype-Tissue Expression Project
CGGA	Chinese Glioma Genome Atlas
GEO	Gene Expression Omnibus
RMA	robust multichip average
FPKM	fragments per kilobase million
TPM	transcripts per kilobase million
OS	Overall survival
IF	Immunofluorescence
DAPI	4’,6-Diamidino2-phenylindole dihydrochloride
CNV	copy number variation
GISTIC	Genomic Identification of Significant Targets in Cance
ESTIMATE	Estimation of Stromal and Immune cells in Malignant Tumor tissues using Expression
TIMER	Tumor Immune Estimation Resource
GSVA	gene set variation analysis
ssGSEA	single sample gene set enrichment analysis
GO	Gene Ontology
KEGG	Kyoto Encyclopedia of Genes and Genomes
GSEA	Gene set enrichment analysis
GDSC	Genomics of Drug Sensitivity in Cancer
IC50	half-maximal inhibitory concentration
CHOL	cholangiocarcinoma
HNSC	head and neck squamous cell carcinoma
KIRC	kidney renal clear cell carcinoma
LAML	acute myeloid leukemia
LIHC	liver hepatocellular carcinoma
PAAD	pancreatic adenocarcinoma
TGCT	testicular germ cell tumors
LGG	lower-grade glioma
GBM	glioblastoma multiforme
IDH	isocitrate dehydrogenase
ACC	adrenocortical carcinoma
BLCA	bladder urothelial carcinoma
BRCA	breast invasive carcinoma
CESC	cervical squamous cell carcinoma and endocervical adenocarcinoma
COAD	colon adenocarcinoma
KIRP	kidney renal papillary cell carcinoma
LUAD	lung adenocarcinoma
LUSC	lung squamous cell carcinoma
MESO	mesothelioma
OV	ovarian serous cystadenocarcinoma
STAD	stomach adenocarcinoma
THCA	thyroid carcinoma
UCEC	uterine corpus endometrial carcinoma
UVM	uveal melanoma
JAK	Janus kinase
STAT	signal transducer and activator of transcription
IL-4	interleukin-4
TTN	titin
EGFR	epidermal growth factor receptor
PTEN	phosphatase and tensin homolog
ATRX	alpha-thalassemia/mental retardation syndrome x-linked chromatin remodeler
CIC	capicua transcriptional repressor
FUBP1	far upstream element binding protein 1
COL6A3	collagen type VI alpha 3 chain
PD-1	programmed cell death 1
PD-L1	Programmed cell death 1 ligand 1
CTLA-4	cytotoxic T-lymphocyte associated protein 4
FAK	focal adhesion kinase
MAPK	Mitogen-Activated Protein Kinase
Erk	Extracellular Regulated Kinase
PI3-K	phosphatidylinositol 3-kinase
Akt	protein kinase B
SAPK	stress-activated MAP kinases
JNK	c-Jun N-terminal kinase
ROC	receiver operating characteristic
AUC	area under the curves
IDH1	isocitrate dehydrogenase [NADP] cytoplasmic
OBSCN	obscurin
Tregs	regulatory T cells
TIDE	Tumor Immune Dysfunction and Exclusion

## References

[1] Ostrom QT Patil N Cioffi G Waite K Kruchko C Barnholtz-Sloan JS. CBTRUS Statistical Report: Primary Brain and Other Central Nervous System Tumors Diagnosed in the United States in 2013-2017. Neuro Oncol (2020) 22(12 Suppl 2):iv1–iv96. doi: 10.1093/neuonc/noaa200 33123732 PMC7596247

[2] Delgado-Martin B Medina MA. Advances in the Knowledge of the Molecular Biology of Glioblastoma and Its Impact in Patient Diagnosis, Stratification, and Treatment. Adv Sci (Weinh) (2020) 7(9):1902971. doi: 10.1002/advs.201902971 32382477 PMC7201267

[3] Wick W Weller M van den Bent M Sanson M Weiler M von Deimling A. MGMT Testing–the Challenges for Biomarker-Based Glioma Treatment. Nat Rev Neurol (2014) 10(7):372–85. doi: 10.1038/nrneurol.2014.100 24912512

[4] Buonfiglioli A Hambardzumyan D. Macrophages and Microglia: The Cerberus of Glioblastoma. Acta Neuropathol Commun (2021) 9(1):54. doi: 10.1186/s40478-021-01156-z 33766119 PMC7992800

[5] Lah TT Novak M Breznik B. Brain Malignancies: Glioblastoma and Brain Metastases. Semin Cancer Biol (2020) 60:262–73. doi: 10.1016/j.semcancer.2019.10.010 31654711

[6] Qi Y Liu B Sun Q Xiong X Chen Q. Immune Checkpoint Targeted Therapy in Glioma: Status and Hopes. Front Immunol (2020) 11:578877. doi: 10.3389/fimmu.2020.578877 33329549 PMC7729019

[7] Weller M Roth P Preusser M Wick W Reardon DA Platten M. Vaccine-Based Immunotherapeutic Approaches to Gliomas and Beyond. Nat Rev Neurol (2017) 13(6):363–74. doi: 10.1038/nrneurol.2017.64 28497804

[8] Prager BC Bhargava S Mahadev V Hubert CG Rich JN. Glioblastoma Stem Cells: Driving Resilience Through Chaos. Trends Cancer (2020) 6(3):223–35. doi: 10.1016/j.trecan.2020.01.009 PMC8779821 32101725

[9] Kuninty PR Bansal R De Geus SWL Mardhian DF Schnittert J van Baarlen J. ITGA5 Inhibition in Pancreatic Stellate Cells Attenuates Desmoplasia and Potentiates Efficacy of Chemotherapy in Pancreatic Cancer. Sci Adv (2019) 5(9):eaax2770. doi: 10.1126/sciadv.aax2770 31517053 PMC6726450

[10] Wang X Che X Yu Y Cheng Y Bai M Yang Z. Hypoxia-Autophagy Axis Induces VEGFA by Peritoneal Mesothelial Cells to Promote Gastric Cancer Peritoneal Metastasis Through an Integrin Alpha5-Fibronectin Pathway. J Exp Clin Cancer Res (2020) 39(1):221. doi: 10.1186/s13046-020-01703-x 33081836 PMC7576728

[11] Dingemans AM van den Boogaart V Vosse BA van Suylen RJ Griffioen AW Thijssen VL. Integrin Expression Profiling Identifies Integrin Alpha5 and Beta1 as Prognostic Factors in Early Stage Non-Small Cell Lung Cancer. Mol Cancer (2010) 9:152. doi: 10.1186/1476-4598-9-152 20565758 PMC2895598

[12] Pantano F Croset M Driouch K Bednarz-Knoll N Iuliani M Ribelli G. Integrin Alpha5 in Human Breast Cancer is a Mediator of Bone Metastasis and a Therapeutic Target for the Treatment of Osteolytic Lesions. Oncogene (2021) 40(7):1284–99. doi: 10.1038/s41388-020-01603-6 PMC7892344 33420367

[13] Blandin AF Noulet F Renner G Mercier MC Choulier L Vauchelles R. Glioma Cell Dispersion is Driven by Alpha5 Integrin-Mediated Cell-Matrix and Cell-Cell Interactions. Cancer Lett (2016) 376(2):328–38. doi: 10.1016/j.canlet.2016.04.007 27063097

[14] Samandari E Visarius T Zingg JM Azzi A. The Effect of Gamma-Tocopherol on Proliferation, Integrin Expression, Adhesion, and Migration of Human Glioma Cells. Biochem Biophys Res Commun (2006) 342(4):1329–33. doi: 10.1016/j.bbrc.2006.02.110 16516856

[15] Zhu H Wang G Zhu H Xu A. ITGA5 is a Prognostic Biomarker and Correlated With Immune Infiltration in Gastrointestinal Tumors. BMC Cancer (2021) 21(1):269. doi: 10.1186/s12885-021-07996-1 33711961 PMC7953822

[16] Liu S Song A Wu Y Yao S Wang M Niu T. Analysis of Genomics and Immune Infiltration Patterns of Epithelial-Mesenchymal Transition Related to Metastatic Breast Cancer to Bone. Transl Oncol (2021) 14(2):100993. doi: 10.1016/j.tranon.2020.100993 33333372 PMC7736716

[17] Liu Z Meng Q Bartek J Jr Poiret T Persson O Rane L. Tumor-Infiltrating Lymphocytes (TILs) From Patients With Glioma. Oncoimmunology (2017) 6(2):e1252894. doi: 10.1080/2162402X.2016.1252894 28344863 PMC5353900

[18] Mermel CH Schumacher SE Hill B Meyerson ML Beroukhim R Getz G. GISTIC2.0 Facilitates Sensitive and Confident Localization of the Targets of Focal Somatic Copy-Number Alteration in Human Cancers. Genome Biol (2011) 12(4):R41. doi: 10.1186/gb-2011-12-4-r41 21527027 PMC3218867

[19] Yoshihara K Shahmoradgoli M Martinez E Vegesna R Kim H Torres-Garcia W. Inferring Tumour Purity and Stromal and Immune Cell Admixture From Expression Data. Nat Commun (2013) 4:2612. doi: 10.1038/ncomms3612 24113773 PMC3826632

[20] Li T Fu J Zeng Z Cohen D Li J Chen Q. TIMER2.0 for Analysis of Tumor-Infiltrating Immune Cells. Nucleic Acids Res (2020) 48(W1):W509–W14. doi: 10.1093/nar/gkaa407 PMC7319575 32442275

[21] Becht E Giraldo NA Lacroix L Buttard B Elarouci N Petitprez F. Erratum to: Estimating the Population Abundance of Tissue-Infiltrating Immune and Stromal Cell Populations Using Gene Expression. Genome Biol (2016) 17(1):249. doi: 10.1186/s13059-016-1113-y 27908289 PMC5134277

[22] Hanzelmann S Castelo R Guinney J. GSVA: Gene Set Variation Analysis for Microarray and RNA-Seq Data. BMC Bioinf (2013) 14:7. doi: 10.1186/1471-2105-14-7 PMC3618321 23323831

[23] Xu L Deng C Pang B Zhang X Liu W Liao G. TIP: A Web Server for Resolving Tumor Immunophenotype Profiling. Cancer Res (2018) 78(23):6575–80. doi: 10.1158/0008-5472.Can-18-0689 30154154

[24] Schreiber RD Old LJ Smyth MJ. Cancer Immunoediting: Integrating Immunity's Roles in Cancer Suppression and Promotion. Science (2011) 331(6024):1565–70. doi: 10.1126/science.1203486 21436444

[25] Zhang M Wang X Chen X Zhang Q Hong J. Novel Immune-Related Gene Signature for Risk Stratification and Prognosis of Survival in Lower-Grade Glioma. Front Genet (2020) 11:363. doi: 10.3389/fgene.2020.00363 32351547 PMC7174786

[26] Jiang P Gu S Pan D Fu J Sahu A Hu X. Signatures of T Cell Dysfunction and Exclusion Predict Cancer Immunotherapy Response. Nat Med (2018) 24(10):1550–8. doi: 10.1038/s41591-018-0136-1 PMC6487502 30127393

[27] Liberzon A Birger C Thorvaldsdottir H Ghandi M Mesirov JP Tamayo P. The Molecular Signatures Database (MSigDB) Hallmark Gene Set Collection. Cell Syst (2015) 1(6):417–25. doi: 10.1016/j.cels.2015.12.004 PMC4707969 26771021

[28] Rosario SR Long MD Affronti HC Rowsam AM Eng KH Smiraglia DJ. Pan-Cancer Analysis of Transcriptional Metabolic Dysregulation Using The Cancer Genome Atlas. Nat Commun (2018) 9(1):5330. doi: 10.1038/s41467-018-07232-8 30552315 PMC6294258

[29] Yu G Wang LG Han Y He QY. Clusterprofiler: An R Package for Comparing Biological Themes Among Gene Clusters. OMICS (2012) 16(5):284–7. doi: 10.1089/omi.2011.0118 PMC3339379 22455463

[30] Mayakonda A Lin DC Assenov Y Plass C Koeffler HP. Maftools: Efficient and Comprehensive Analysis of Somatic Variants in Cancer. Genome Res (2018) 28(11):1747–56. doi: 10.1101/gr.239244.118 PMC6211645 30341162

[31] Gu Z Eils R Schlesner M. Complex Heatmaps Reveal Patterns and Correlations in Multidimensional Genomic Data. Bioinformatics (2016) 32(18):2847–9. doi: 10.1093/bioinformatics/btw313 27207943

[32] Pearce EL Pearce EJ. Metabolic Pathways in Immune Cell Activation and Quiescence. Immunity (2013) 38(4):633–43. doi: 10.1016/j.immuni.2013.04.005 PMC3654249 23601682

[33] Stupp R Mason WP van den Bent MJ Weller M Fisher B Taphoorn MJ. Radiotherapy Plus Concomitant and Adjuvant Temozolomide for Glioblastoma. N Engl J Med (2005) 352(10):987–96. doi: 10.1056/NEJMoa043330 15758009

[34] Huang R Rofstad EK. Integrins as Therapeutic Targets in the Organ-Specific Metastasis of Human Malignant Melanoma. J Exp Clin Cancer Res (2018) 37(1):92. doi: 10.1186/s13046-018-0763-x 29703238 PMC5924434

[35] Aksorn N Chanvorachote P. Integrin as a Molecular Target for Anti-Cancer Approaches in Lung Cancer. Anticancer Res (2019) 39(2):541–8. doi: 10.21873/anticanres.13146 30711928

[36] Li M Wang Y Li M Wu X Setrerrahmane S Xu H. Integrins as Attractive Targets for Cancer Therapeutics. Acta Pharm Sin B (2021) 11(9):2726–37. doi: 10.1016/j.apsb.2021.01.004 PMC8463276 34589393

[37] Asl ER Amini M Najafi S Mansoori B Mokhtarzadeh A Mohammadi A. Interplay Between MAPK/ERK Signaling Pathway and MicroRNAs: A Crucial Mechanism Regulating Cancer Cell Metabolism and Tumor Progression. Life Sci (2021) 278:119499. doi: 10.1016/j.lfs.2021.119499 33865878

[38] Cohen AL Colman H. Glioma Biology and Molecular Markers. Cancer Treat Res (2015) 163:15–30. doi: 10.1007/978-3-319-12048-5_2 25468223

[39] Chen J Chen T Zhu Y Li Y Zhang Y Wang Y. circPTN Sponges miR-145-5p/miR-330-5p to Promote Proliferation and Stemness in Glioma. J Exp Clin Cancer Res (2019) 38(1):398. doi: 10.1186/s13046-019-1376-8 31511040 PMC6737709

[40] Kita D Takino T Nakada M Takahashi T Yamashita J Sato H. Expression of Dominant-Negative Form of Ets-1 Suppresses Fibronectin-Stimulated Cell Adhesion and Migration Through Down-Regulation of Integrin Alpha5 Expression in U251 Glioma Cell Line. Cancer Res (2001) 61(21):7985–91. 11691823

[41] Sampson JH Gunn MD Fecci PE Ashley DM. Brain Immunology and Immunotherapy in Brain Tumours. Nat Rev Cancer (2020) 20(1):12–25. doi: 10.1038/s41568-019-0224-7 31806885 PMC7327710

[42] Friebel E Kapolou K Unger S Nunez NG Utz S Rushing EJ. Single-Cell Mapping of Human Brain Cancer Reveals Tumor-Specific Instruction of Tissue-Invading Leukocytes. Cell (2020) 181(7):1626–42 e20. doi: 10.1016/j.cell.2020.04.055 32470397

[43] Sa JK Chang N Lee HW Cho HJ Ceccarelli M Cerulo L. Transcriptional Regulatory Networks of Tumor-Associated Macrophages That Drive Malignancy in Mesenchymal Glioblastoma. Genome Biol (2020) 21(1):216. doi: 10.1186/s13059-020-02140-x 32847614 PMC7448990

[44] Pombo Antunes AR Scheyltjens I Duerinck J Neyns B Movahedi K Van Ginderachter JA. Understanding the Glioblastoma Immune Microenvironment as Basis for the Development of New Immunotherapeutic Strategies. Elife (2020) 9. doi: 10.7554/eLife.52176 PMC7000215 32014107

[45] Sepulveda-Sanchez JM Vaz MA Balana C Gil-Gil M Reynes G Gallego O. Phase II Trial of Dacomitinib, a Pan-Human EGFR Tyrosine Kinase Inhibitor, in Recurrent Glioblastoma Patients With EGFR Amplification. Neuro Oncol (2017) 19(11):1522–31. doi: 10.1093/neuonc/nox105 PMC5737732 28575464

[46] Reardon DA Desjardins A Vredenburgh JJ O'Rourke DM Tran DD Fink KL. Rindopepimut With Bevacizumab for Patients With Relapsed EGFRvIII-Expressing Glioblastoma (ReACT): Results of a Double-Blind Randomized Phase II Trial. Clin Cancer Res (2020) 26(7):1586–94. doi: 10.1158/1078-0432.CCR-18-1140 32034072

[47] Blandin AF Cruz Da Silva E Mercier MC Glushonkov O Didier P Dedieu S. Gefitinib Induces EGFR and Alpha5beta1 Integrin Co-Endocytosis in Glioblastoma Cells. Cell Mol Life Sci (2021) 78(6):2949–62. doi: 10.1007/s00018-020-03686-6 PMC11073190 33151388

[48] Farber K Synowitz M Zahn G Vossmeyer D Stragies R van Rooijen N. An Alpha5beta1 Integrin Inhibitor Attenuates Glioma Growth. Mol Cell Neurosci (2008) 39(4):579–85. doi: 10.1016/j.mcn.2008.08.005 18804537

