# Supplementary figures and images for: ITGA5 Is a Novel Oncogenic Biomarker and Correlates With Tumor Immune Microenvironment in Gliomas
Source: Front Oncol. 2022 Mar 18;12:844144. doi: 10.3389/fonc.2022.844144 (PMC8971292; doi:10.3389/fonc.2022.844144)

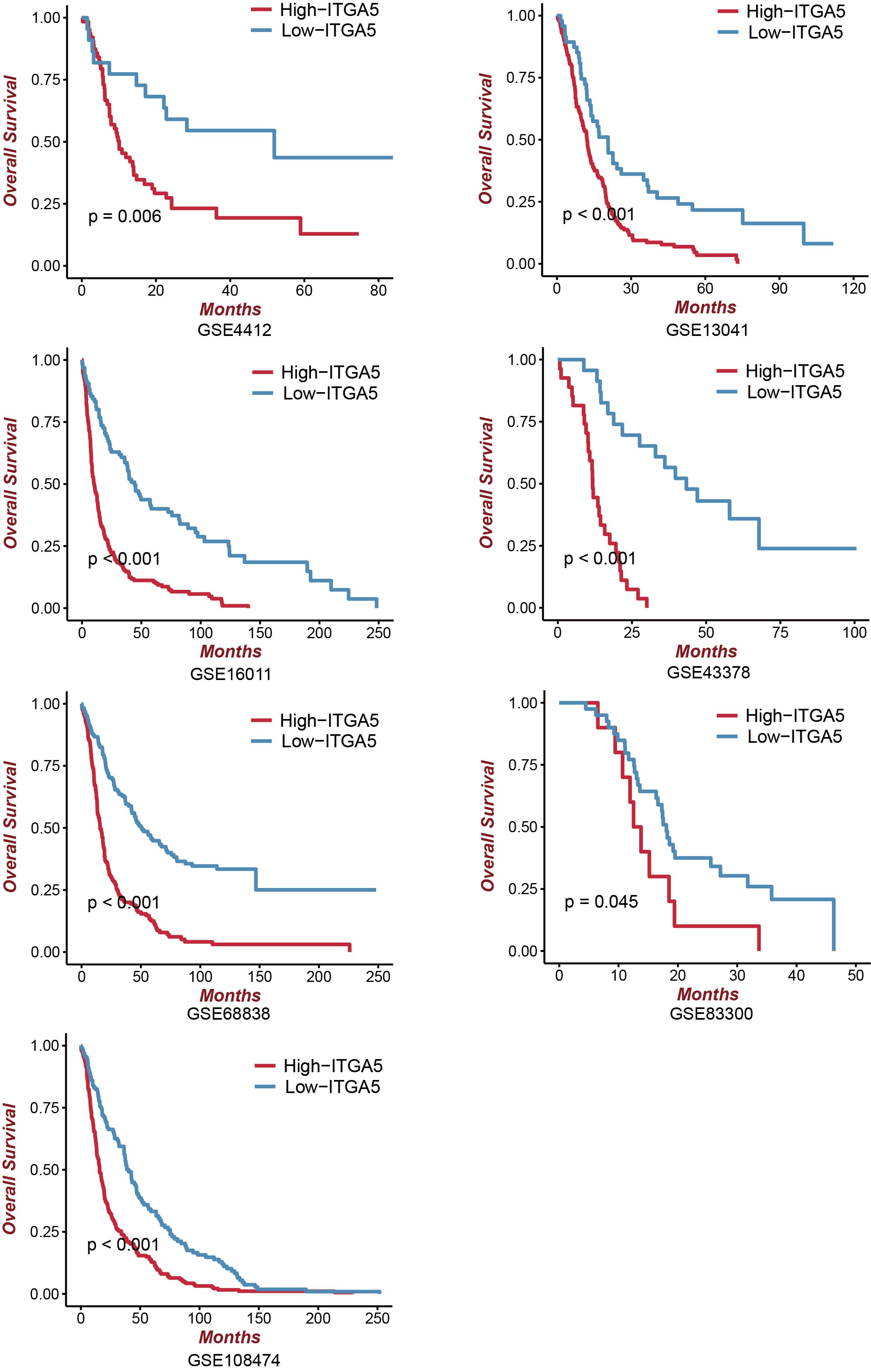

Supplement: Supplementary Figure 1 — The Kaplan–Meier curves of ITGA5 in 7 independent GEO datasets. [file Image_1.jpeg]

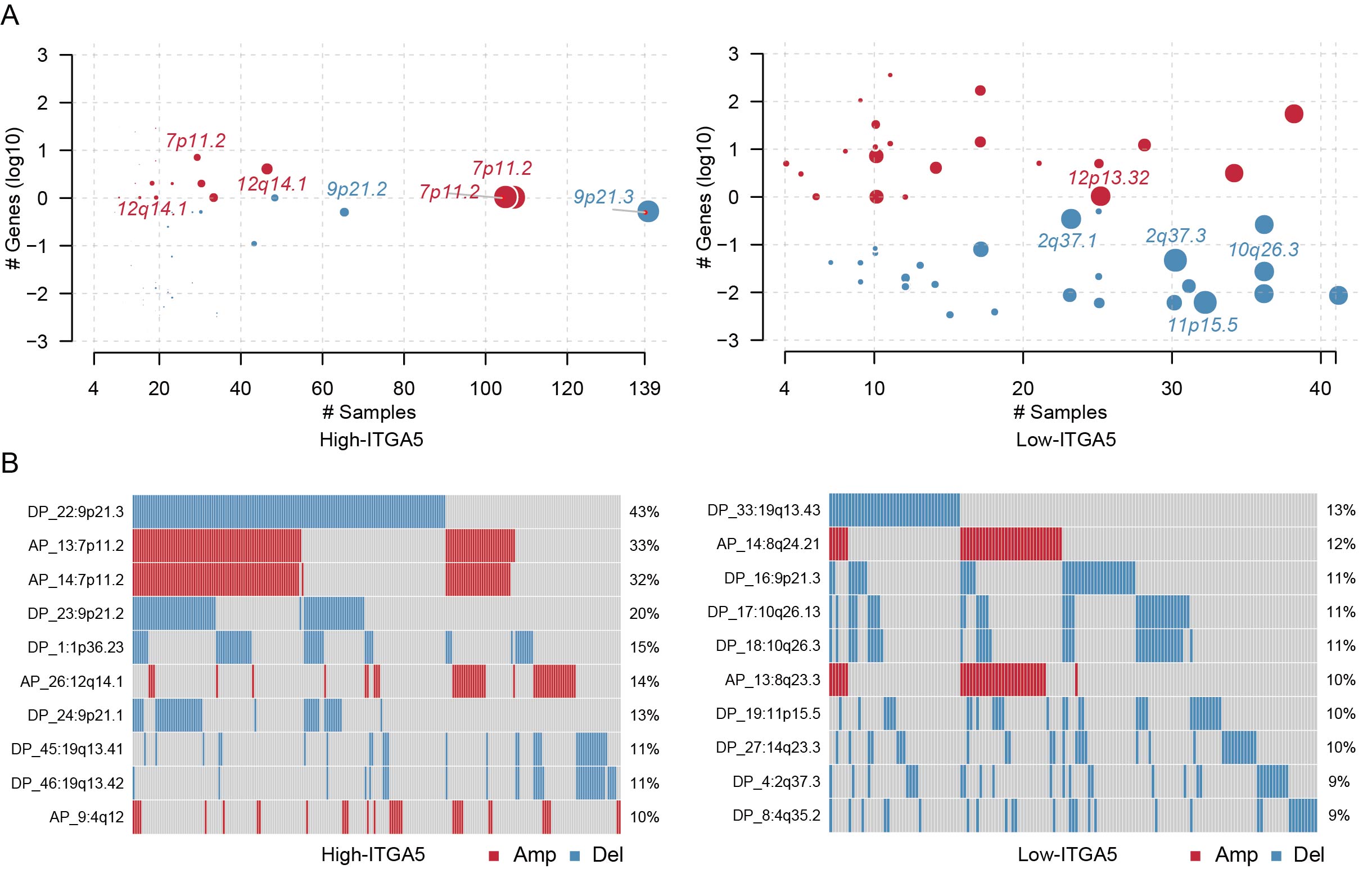

Supplement: Supplementary Figure 2 — The genomic alterations in high-ITGA5 and low-ITGA5 groups. (A) Amplification and deletions in gliomas with high and low ITGA5 expression. (B) The detailed amplification and deletion of chromosome copy number variation in gliomas with high and low ITGA5 expression. [file Image_2.jpeg]

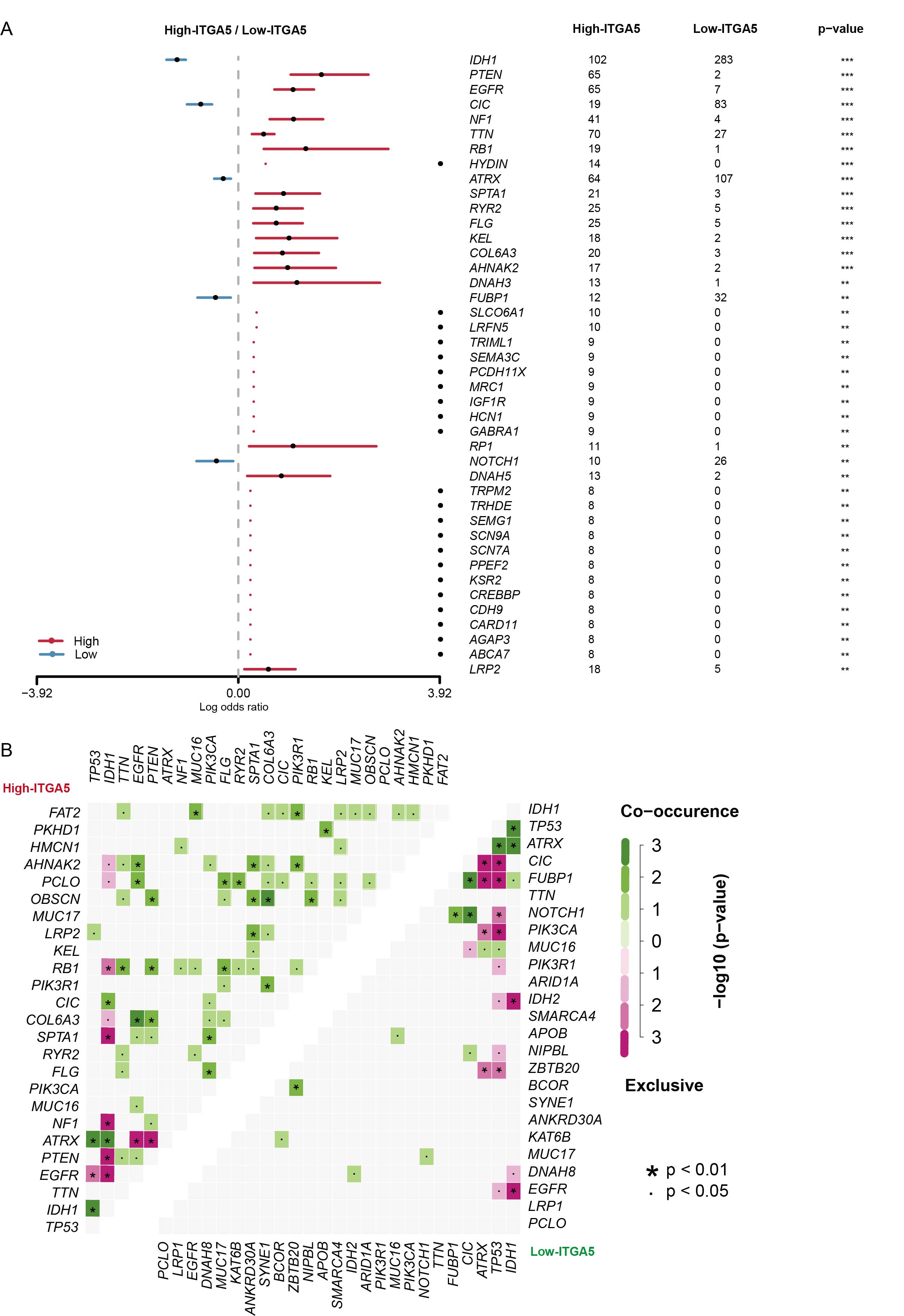

Supplement: Supplementary Figure 3 — The somatic mutations in high-ITGA5 and low-ITGA5 groups. (A) The forest plot illustrates the difference in mutation patterns between high-ITGA5 and low-ITGA5 groups. (B) The heatmap presents the somatic interaction in gliomas with high and low ITGA5 levels. [file Image_3.jpeg]

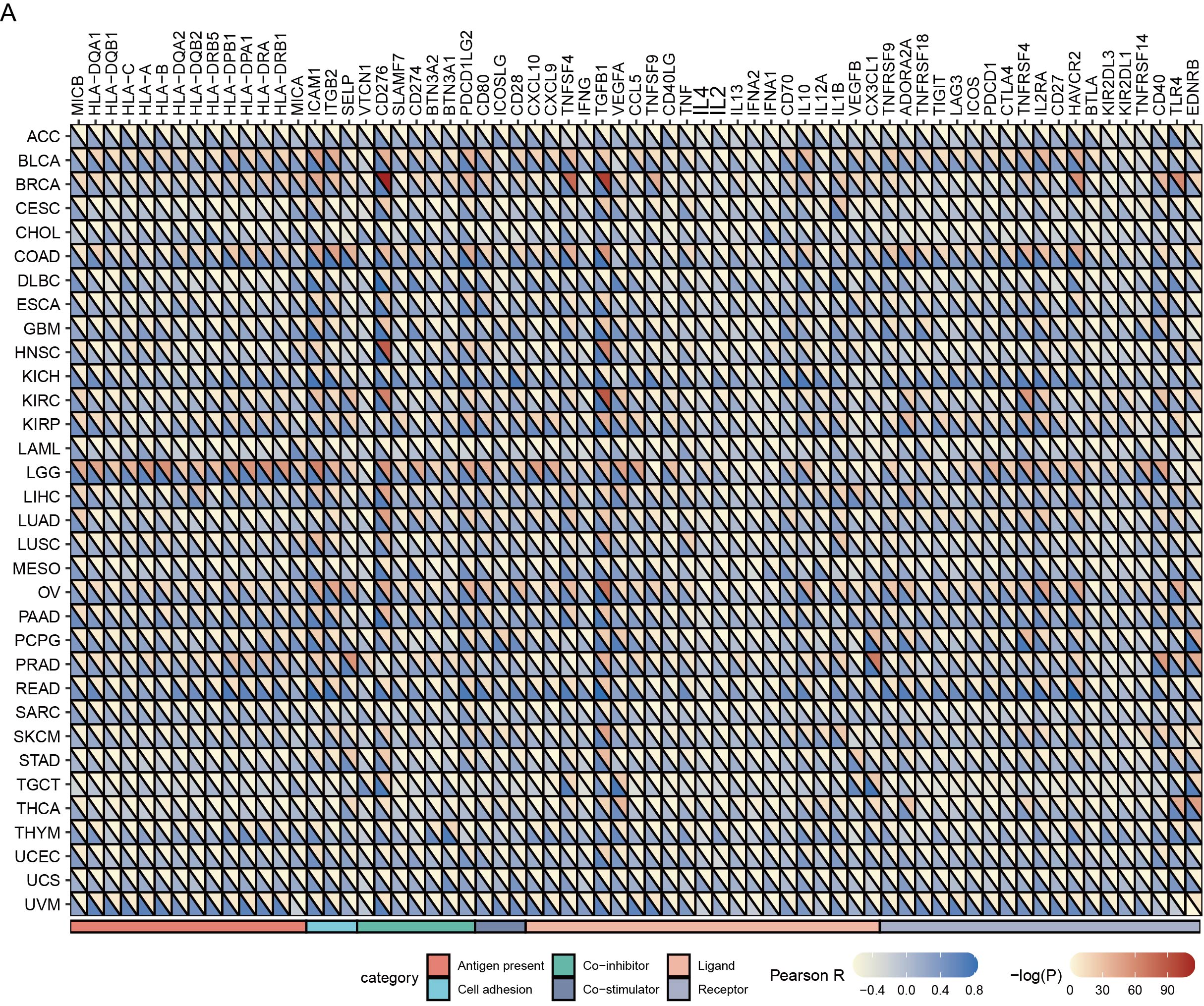

Supplement: Supplementary Figure 4 — The correlation between ITGA5 expression and recognized immune checkpoints in pan-cancer. [file Image_4.jpeg]

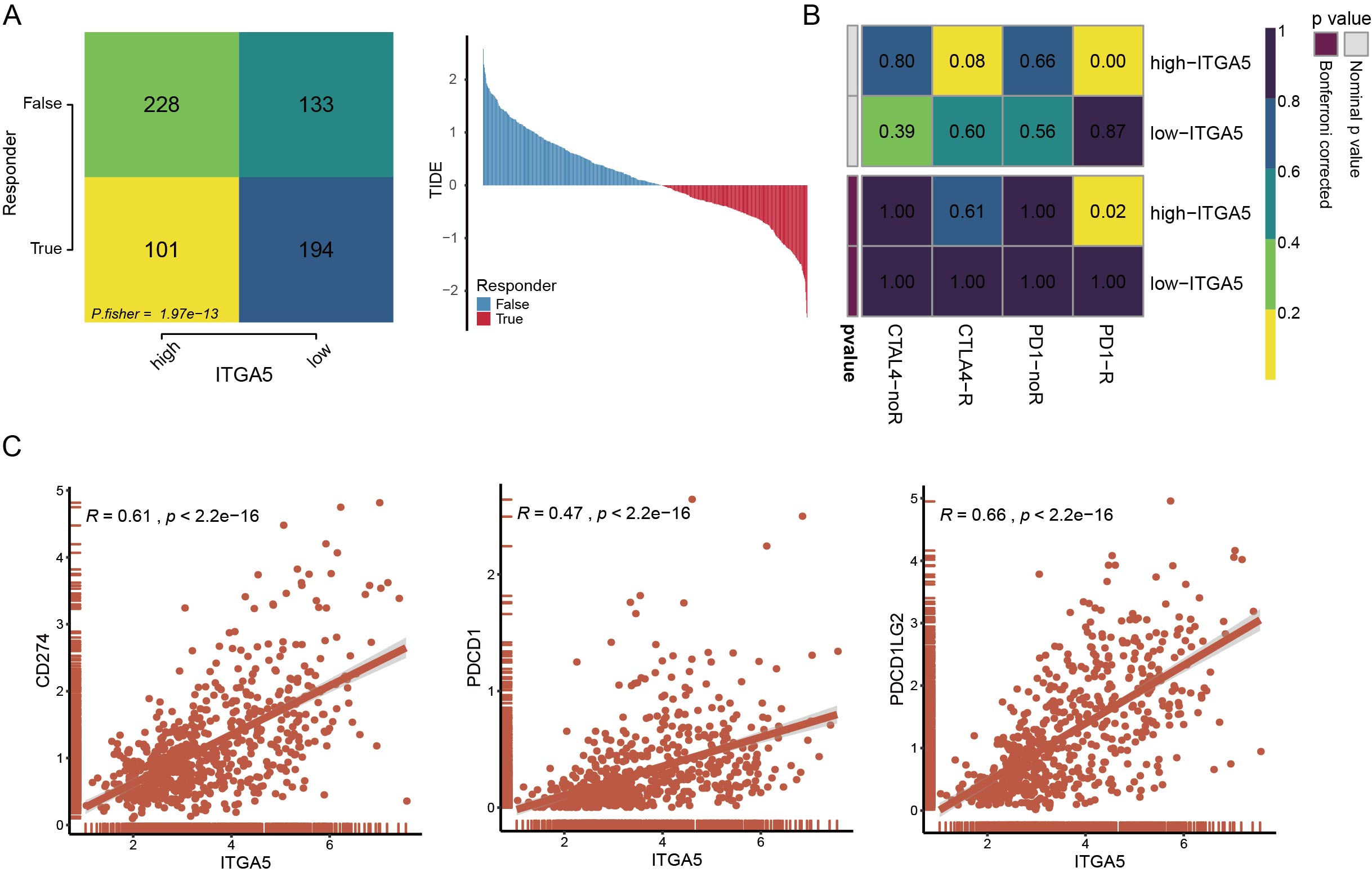

Supplement: Supplementary Figure 5 — The evaluation of the sensitivity of ITGA5 to immunotherapy. (A) TIDE predicting the immunotherapy responses related to ITGA5 in gliomas. (B) Submap analysis of ITGA5 expression levels in gliomas. (C) The corrplot of the correlation between ITGA5 expression and immune checkpoints. [file Image_5.jpeg]
